# Fluoroquinolone resistance of *Staphylococcus epidermidis* isolated from healthy conjunctiva and analysis of their mutations in quinolone-resistance determining region

**DOI:** 10.1186/s13756-020-00841-3

**Published:** 2020-11-04

**Authors:** Jung Youb Kang, Woonhyoung Lee, Gwang Myeong Noh, Bo Hyun Jeong, Indal Park, Sang Joon Lee

**Affiliations:** 1 Department of Ophthalmology, Kosin University College of Medicine, 262 Gamchun-ro, Seo-gu, Busan, South Korea; 2 Department of Laboratory Medicine, Kosin University College of Medicine, Busan, South Korea; 3 Department of Microbiology, Kosin University College of Medicine, Busan, South Korea

**Keywords:** *Staphylococcus epidermidis*, Quinolone resistance, QRDR, Mutation, Conjunctiva, Microbes

## Abstract

**Background:**

*Staphylococcus epidermidis* is the most common pathogen in postoperative endophthalmitis and causes various infectious eye diseases. However, there is very little information on fluoroquinolone antibiotic resistance to *S. epidermidis* identified in conjunctival microbe and analysis of related genes. Here, the authors investigated the rate of resistance to fluoroquinolones of *Staphylococcus epidermidis* isolated from normal conjunctival microbes and mutations in the quinolone-resistance determining region (QRDR).

**Methods:**

377 eye samples from 187 patients who underwent intravitreal injection and cataract surgery were included. Specimens were taken from the bilateral lower conjunctival sacs using a cotton swab and cultured. The cultures were identified using MALDI-TOP MS and *gyrA, gyrB, parC*, and *parE* gene mutations of QRDR were confirmed by DNA extraction from resistant strains of *S. epidermidis* with a micro-dilution method using ciprofloxacin, levofloxacin, and moxifloxacin*.*

**Results:**

The culture positive rate was 61.8% (231) for 374 eye samples. Of the 303 total strains cultured, *S. epidermidis* was the most common with 33.7% (102). Ten types of gene mutations were observed in the resistant *S. epidermidis* of 21 strains. One-point mutation was observed mainly in *gyrA* and *parC*, and a small number of mutations were observed in *parE* in the form of a double point mutations. When there were multiple point mutations in both *gyrA* and *parC*, the highest minimum inhibitory concentration was observed.

**Conclusions:**

The quinolone resistance rate of *S. epidermidis* increased in comparison with previous studies, and resistant *S. epidermidis* showed mostly QRDR mutations, which were mainly found in *gyrA* and *parC*, and showed strong resistance when mutated in both genes.

## Background

Postoperative endophthalmitis is a fatal complication that can lead to blindness, and it is essential to use appropriate antibiotics based on the causative microorganisms. The most common causative strain of postoperative endophthalmitis has been thought to be *Staphylococcus epidermidis*, a type of coagulase-negative staphylococci (CNS) [1–3]. Most of the isolates cultured from postoperative endophthalmitis come from conjunctival flora [4]. With this in mind, it is important to investigate the antibiotic susceptibility of *S. epidermidis.* Although *S. epidermidis* is the most common microorganism among conjunctival microbes, studies on the composition of the conjunctival microbes including *S. epidermidis* and their fluoroquinolone resistance are insufficient [5–7].

Fluoroquinolone, which is frequently used as a topical antibiotic agent in ophthalmology, primarily inhibits DNA gyrase (topoisomerase II) and topoisomerase lV, which are essential enzymes in bacteria [8–10]. This kind of antibiotics cover broad spectrum bacteria, including most aerobic gram-negative and gram-positive bacteria, and possess low toxicity and good ocular surface penetration characteristics [11, 12]. In the 1990s, ciprofloxacin 0.3% and ofloxacin 0.3% were introduced, and were widely used for the treatment and prevention of ocular bacterial infection. Levofloxacin 0.5%, gatifloxacin 0.3%, and moxifloxacin 0.5% were introduced after 2000. Both gatifloxacin and moxifloxacin, which are 8-methoxyfluoroquinolones, interfere with bacterial DNA gyrase and topoisomerase IV, which are enzymes involved in DNA replication [13, 14]. Fluoroquinolones are one of the most frequently used eyedrops in the prophylactic treatment of postoperative endophthalmitis. The incidence of in vitro resistance to these fluoroquinolones has been reported to be increasing [15–17]. Therefore, it is important to characterize the normal ocular bacterial flora and to determine antibiotic susceptibility patterns to select appropriate antibiotics for prophylaxis of postoperative endophthalmitis. There exist three research papers regarding fluoroquinolone resistance of conjunctival microbes from 1999, 2001, and 2009. The studies published in 2001 and 2009 reported that CNS resistance was increasing compared to previous reports [5–7]. Unfortunately, no studies on conjunctival normal flora have been reported for about 10 years, since 2009 [5–7].

In *S. epidermidis*, DNA gyrase and topoisomerase lV each have two subunits, GyrA and GyrB, and ParC and ParE, respectively. Most resistant bacteria show mutations in specific regions of these four subunits, the quinolone-resistance determining region (QRDR). The QRDR mutations of fluoroquinolone-resistant *S. epidermidis* cultured on the surface of the eyeball have not been studied [18, 19]. It is important to characterize the normal bacterial flora in ocular surface and to determine the antibiotic susceptibility patterns to select appropriate antibiotics for prophylaxis of postoperative endophthalmitis.

Therefore, in this study, we investigated the composition of the normal conjunctival flora and the rate of resistance to the three fluoroquinolones (ciprofloxacin, levofloxacin, moxifloxacin) of *S. epidermidis*, considered to be the most common causative agent of postoperative endophthalmitis, and the gene mutation pattern of the QRDR of resistant *S. epidermidis*.

## Methods

### Research subjects

A total of 187 patients (374 eye samples), from Kosin University Gospel Hospital between May 1, 2016 and September 31, 2017, were included. Of these, 120 (240 eyes) were scheduled for intravitreal injection and 67 (134 eyes) were scheduled for cataract surgery. Patients with the following conditions that were able to affect the conjunctival flora were excluded from the study: patients with a history of surgery, hospitalization, or use of systemic antibiotics within 3 months, patients who have used intraocular surgery or vitreous injection, glaucoma eye drops, or antimicrobial eye drops within 3 months.

### Sample collection and storage

Samples were collected using a polyester tipped swab (23-400-122, Fisherbrand™, USA) from the lower conjunctival sac prior to the instillation of an anesthetic agent without the use of a prophylactic antimicrobial agent before the operation. Immediately after inoculation on a 5% blood agar plate, the cells were cultured for up to 7 days in an incubator (Water-Jacketed CO2 Incubator, Forma Scientific, Inc., USA). When the bacteria grew, they were sorted based on the shape of the colony, and stored in Eppendorf tubes. Bacteria were harvested from 1 to 2 colonies in 1 mL of a mixture of glycerol and brucella broth (3:7) and stored in a − 70 °C cryogenic refrigerator.

### Identification of bacteria

Frozen samples were thawed and inoculated on 5% blood agar plates using a 10 μL loop (SPL Life Sciences, Korea). Subsequently, the cells were subcultured once to identify the bacteria and conduct antimicrobial susceptibility tests. Identification of the bacteria was performed using Matrix assisted laser desorption/ionization time-of-flight mass spectrometry (MALDI-TOF MS, Bruker Daltonics GmbH, Germany).

### Fluoroquinolone susceptibility test of *S. epidermidis*

The antimicrobial susceptibility test for the quinolone formulation was performed using a micro-dilution test using a liquid medium (tryptic soy broth: TSB) and a 96-well plate. The quinolone preparation was made by diluting ciprofloxacin 200 mg/100 mL (Ciprobay®, Bayer, Germany), Levofloxacin 750 mg (Cravit®, Jeilpharm, Korea), moxifloxacin hydrochloride 436.8 mg/250 mL (Avelox®, Bayer, Germany). The results were interpreted according to the Clinical and Laboratory Standards Institute (CLSI M100 S26) standards, and the concentrations of the tolerance standards are shown in Table 1.

**Table 1:** Clinical and Laboratory Standards Institute Performance Standards for dilution antimicrobial susceptibility tests

Antimicrobial agent	MIC (μg/mL) interpretive standard		
	Susceptible	Intermediate	Resistant
Ciprofloxacin	≤ 1	2	≥ 4
Levofloxacin	≤ 1	2	≥ 4
Moxifloxacin	≤ 0.5	1	≥ 2

*MIC* minimal inhibitory concentration

For ciprofloxacin and levofloxacin, the highest concentration was 32 μg/mL, the lowest concentration was 0.062 μg/mL; the highest concentration of moxifloxacin was 16 μg/mL, and the lowest concentration was 0.032 μg/mL. The bacterial dilution was incubated with tryptic soy agar (TSA) by thawing the stored frozen strain, and incubated for 24 h. The resulting colonies were mixed well with sterile saline and adjusted to McFarland standard 0.5. The antibiotic solution was diluted by concentration, using cation adjusted Muller Hinton broth (CAMHB), and a strain solution diluted in 0.85% sterile physiological saline was divided into 96-well plates.

**Fig. 1.**
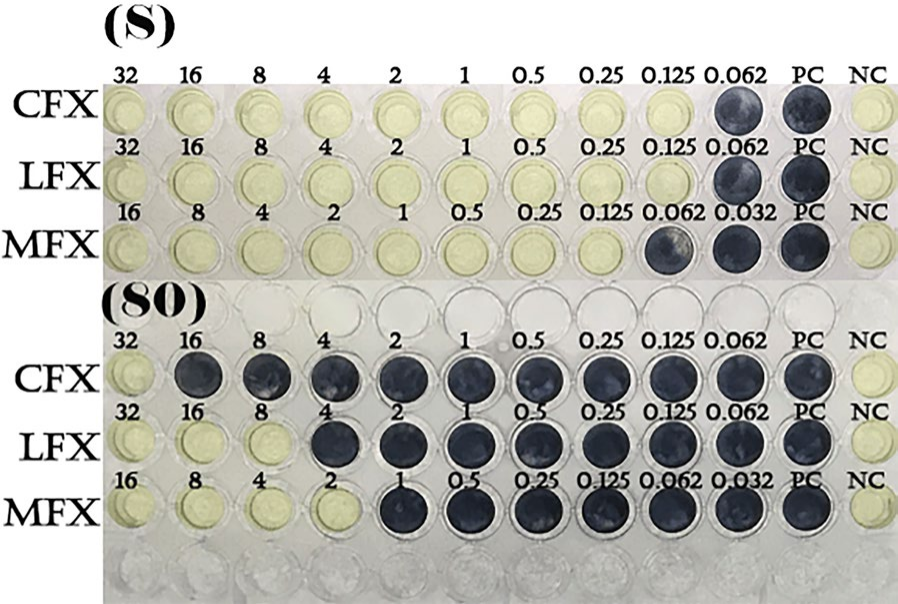
Representative picture of a micro-dilutional antibiotic sensitivity test for *Staphylococcus epidermidis.* *CFX* ciprofloxacin, *LFX* levofloxacin, *MFX* moxifloxacin, *S* standard microorganism (susceptible strain), *80* sample number (resistant strain), *PC* positive control (CAMBH 80 μL + bacterial solution 10 μL), *NC* negative control (CAMBH 80 μL + antibiotics solution 10 μL), Small numbers above each well: concentration of antibiotics

80 μL of liquid medium (CAMHB), 10 μL of diluted antibiotic, and 10 μL of strain solution were dispensed and cultured for 24 h. Subsequently, 5 μL (10 mg/mL) of 3-(4,5-dimethylthiazol-2yl)-2,5-diphenyl tetrazolium bromide (MTT, Sigma, USA) solution was added and observed to change color after 2 h. The lowest inhibitory concentration was determined as the minimum inhibitory concentration (MIC, μg/mL). As a positive control group, medium and bacterial dilution were used; for a negative control, medium alone was used (Fig. 1).

### Sequence analysis of polymerase chain reaction (PCR) and QRDR

**Fig. 2.**
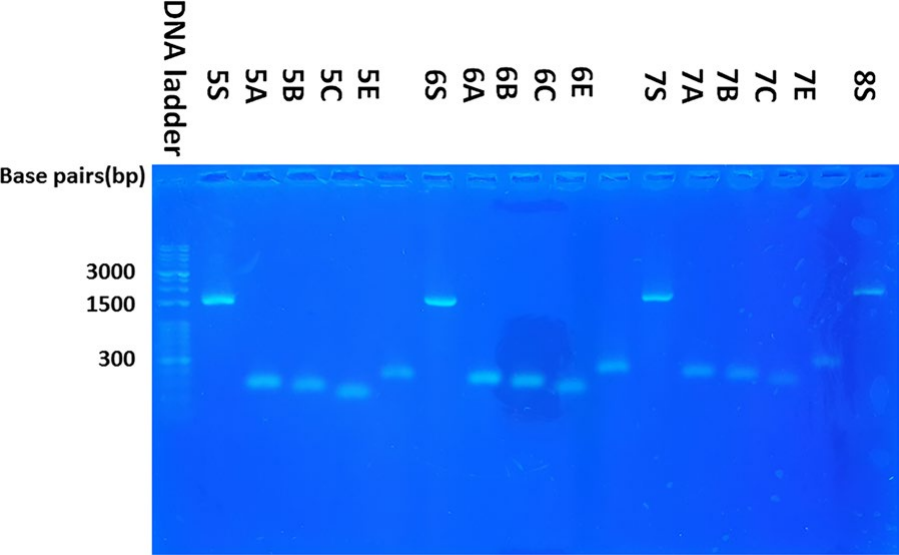
Representative picture of a 1.2% agarose gel electrophoresis of polymerase chain reaction (PCR) products. Arabic numbers: sample numbers, S: 16 s rRNA, A: *gyrA*, B: *gyrB*, C: *parC*, E: *parE*

In order to analyze the mutations of the QRDR sequence, 21 *S. epidermidis* strains with quinolone resistance confirmed were cultured in TSB for 1 day. In order to perform PCR, 0.5 μL of the TSB was taken. Next, 2 μL PCR primer (forward 1 μL, reverse 1 μL), 10 μL TOP real™ qPCR 2X Premix (Enzynomics, Korea), and 8 μL diethyl pyrocarbonate (DEPC) were mixed with water to perform PCR. The PCR primer for QRDR sequencing used the base sequence of *S. epidermidis* RP62A used by Yamada et al. [19]; 16S rRNA sequencing was also performed to confirm that it was an *S. epidermidis* strain. The sequence of the primer used is shown in Table 2. PCR was performed at 94 °C, 30 s, 55 °C, 30 s, 72 °C, and 1 min for 30 cycles, and the PCR product size was confirmed using 1.2% agarose gel electrophoresis (Fig. 2). PCR products were sequenced using DNA purification and an ABI PRISM 3730XL Analyzer (96 capillary type, Thermo Fisher Scientific, USA). For the 16S rRNA sequence, the bacteria were identified using the nucleotide Basic Local Alignment Search Tool (BLAST) of the National Center for Biotechnology Information (NCBI, Additional file 1: Table S1). For the QRDR sequence, SnapGene version 4.2 (GSL Biotech, Canada) and Genetyx version 6 (GENETYX CORP, Japan) were used to analyze the reference sequence (SE RP62A) and nucleotide homology.

**Table 2:** Primers used in the study

Target gene	Primer sequence (5′ to 3′)	Product size (bp)
*gyrA* (FOR)	ATGCGTGAATCATTCTTAGACTATGC	284
*gyrA* (REV)	GAGCCAAAGTTACCTTGACC	
*gyrB* (FOR)	CAGCATTAGACGTTTCAAG	251
*gyrB* (REV)	CCAATACCCGTACCAAATGC	
*parC* (FOR)	TCGCAATGTATTCAAGTGGG	197
*parC* (REV)	ATCGTTATCGATACTACCATT	
*parE* (FOR)	AAGCTCAACAAGCACGCGAGGCTG	324
*parE* (REV)	TTAAAGTCAGTACCAACACCAGCAC	

Nucleotide positions are indicated according to GenBank sequence number NC 002976 (*Staphylococcus epidermidis* RP62A)

## Results

### Profile of isolated microorganisms

The positive rate of culture was 61.76%, or 231 out of 374 eye samples; 26.84%, or 62 of the 231 eye samples cultured two or more strains. There was 21.39%, or 40 out of 187 person cases in which more than one identical strains isolated from both eyes. The culture rates of gram-positive and -negative bacteria were 72.94% and 27.06%, respectively, and the composition is shown in Tables 3 and 4. Among gram-positive bacteria, *S. epidermidis* was the most common isolate (102 eye samples, 33.66%), followed by *Corynebacterium* spp. (54 eye samples, 17.82%), of which 45 were *Corynebacterium macginleyii*. The third most cultured strain was CNS, excluding *S. epidermidis* (30 eye samples, 9.9%), followed by *Enterococcus faecalis* and *S. aureus* (10 eye samples, 3.3%).

**Table 3:** Profile of microorganisms isolated from lower conjunctival sac of eyes undergoing cataract surgery and intravitreal injection

Gram + bacteria (n = 221)	Number of isolates (%)	Gram—bacteria (n = 82)	Number of isolates (%)
*Staphylococcus epidermidis*	102 (33.7%)	*Ochrobactrum* spp.	33 (10.9%)
*Corynebacterium* spp.	54 (17.8%)	*Pseudomonas* spp.	11 (3.6%)
Other CNS	30 (9.9%)	*Achromobacter* spp.	7 (2.3%)
*Enterococcus faecalis*	10 (3.3%)	*Brevundimonas* spp.	7 (2.3%)
*Staphylococcus aureus*	10 (3.3%)	*Enterobacter aerogenes*	4 (1.3%)
*Micrococcus luteus*	6 (2%)	*Roseomonas gilardii*	4 (1.3%)
*Propionibacterium avidum*	4 (1.3%)	*Bordetella hinzii*	3 (1%)
*Kocuria* spp.	3 (1%)	*Acinetobacter baylyi*	2 (0.7%)
*Bacillus pumilus*	1 (0.3%)	*Delftia acidovorans*	2 (0.7%)
*Dermabacter hominis*	1 (0.3%)	*Proteus mirabilis*	2 (0.7%)
		*Sphingomonas paucimobilis*	2 (0.7%)
		*Stenotrophomonas maltophilia*	2 (0.7%)
		*Cupriavidus pauculus*	1 (0.3%)
		*Moraxella* spp.	1 (0.3%)
		*Morganella morganii*	1 (0.3%)

**Table 4:** Susceptibility rates of *Staphylococcus epidermidis* (n = 82) to 3 fluoroquinolone antibiotics

	Ciprofloxacin, n (%)	Levofloxacin, n (%)	Moxifloxacin, n (%)
Susceptible	55 (67.1%)	55 (67.1%)	61 (74.4%)
Intermediate	8 (9.8%)	7 (8.5%)	7 (8.5%)
Resistant	19 (23.2%)	20 (24.4%)	14 (17.1%)

Among gram-negative bacteria, *Ochrobactrum* spp. were the most common isolate (33 eye samples, 10.89%), and *Ochrobactrum intermedium* was cultured in 23 of 33 eye samples. The second most cultured strain was *Pseudomonas* spp. (11 eye samples, 3.63%), followed by *Achromobacter* spp. (7 eye samples, 2.31%), and *Brevundimonas* spp. (7 eye samples, 2.31%).

#### *S. epidermidis* fluoroquinolone susceptibility test

Of the 102 *S. epidermidis* strains, antibiotic susceptibility tests were performed on 82 strains owing to the loss of 20 strains due to storage problems, and the results are shown in Additional file 1: Table S2. Table 4 shows the ciprofloxacin, levofloxacin, and moxifloxacin susceptibility results for 21 bacteria that showed resistance to the fluoroquinolones.

### QRDR gene mutation pattern

A total of 10 types of QRDR gene mutation patterns were observed (Table 5). Point mutations were observed mainly in *gyrA* and *parC*, and the most common ones were Ser84Phe in GyrA and Ser80Tyr in ParC. A small number of mutations were also observed in *parE*, with double point mutations mainly occurring in Lys402Arg, Asn404Ile, Lys403Thr, Asn404Asp, and Lys402Arg. No strain showing *gyrB* mutation was found, and in one strain, no mutation was observed in the QRDR (type 10).

**Table 5:** Mutations in the quinolone resistance determining region (QRDR) of *gyrA*, *gyrB*, *parC* and *parE* in 21 strains of *Staphylococcus epidermidis*

Mutation type	No. of isolates	Mutation			
		*gyrA*	*gyrB*	*parC*	*parE*
1	7	Ser84Phe	–	Ser80Tyr	–
2	4	Ser84Phe	–	Ser80Phe	–
3	3	Ser84Phe	–	Ser80Phe + Asp84Val	–
4	1	Ser84Tyr	–	Ser80Ile	–
5	1	Ser84Tyr	–	Ser80Ile	Lys402Arg + Asn404Ile
6	1	Ser84Phe	–	Ser80Phe + Asp84Val	Lys403Thr + Asn404Asp
7	1	Ser84Phe	–	Ser80Phe + Asp84Val	Lys402Arg + Lys403Arg
8	1	Ser84Tyr + Glu88Lys	–	Ser80Phe + Asp84Tyr	–
9	1	Ser84Phe	–	Ser80Phe	Asp434Val
10	1	–	–	–	–

## Discussion

As a result of cultivating samples collected from the lower conjunctival sac, most (72.94%) of the normal conjunctival flora comprised gram-positive bacteria. The most isolated *S. epidermidis* with a single strain was similar to the previous domestic report [5, 6]. However, some differences from previous domestic and foreign studies were observed in the composition of normal flora. The first difference was that the cultivation rate of *S. epidermidis* among gram-positive bacteria was significantly reduced. In this study, the culture rate of *S. epidermidis* was 33.7% of the total cultured strains, which is a significantly lower culture rate compared to 60.6% in 2009 domestic reports [7] and 56.4% in 2013 Midwest reports [20]. The cause of this phenomenon is probably related to the increased use of topical antibiotics, that is, the use of fluoroquinolone as an ophthalmic antibiotic. The most commonly prescribed topical antibiotic after cataract surgery reported by the Survey of Korean Society of Cataract and Refractive Surgery was fluoroquinolone, which increased from 78% in 2007 to 85% in 2012. Among them, the frequency of prescriptions for moxifloxacin, a third-generation fluoroquinolone, rose sharply from 11% in 2007 to 44% in 2012 [21, 22]. The second difference was that the culture rate of *Corynebacterium* spp. among the gram-positive bacteria increased significantly. In previous domestic reports [5–7], *Corynebacterium* spp. accounted for less than 10% of all strains of Gram-positive bacteria, but in this study, it was observed with a high culture rate of 17.8% compared to 3.3% of Staphylococcus aureus culture (Table 3).

Among the gram-negative bacteria, there were notable differences compared to previous studies. In previous domestic studies [5–7], *Pseudomonas* spp. was observed at a high rate, but in this study, *Ochrobactrum* spp. was cultured at the highest rate. *Ochrobactrum intermedium* was observed in 23 of 33 eyes with *Ochrobactrum* spp, and this strain was not well identified by previous biochemical research methods, but is known to be identified by MALDI-TOF mass spectrometry, a commonly-used identification method [23]. In other words, in previous domestic studies [5–7], it was thought that it was difficult to identify this strain because bacteria were mainly identified using biochemical methods. *Ochrobactrum intermedium* has been reported to cause endophthalmitis in relation to metal ocular foreign bodies [24], and infections other than the eye are rare, so there have not been many studies [25]. It is noteworthy that, despite the strains that were not mentioned at all as constitutive gram-negative bacteria in the previous conjunctival normal flora study in Korea, they were the third most cultivated in this study after *S. epidermidis* and *C. macginleyi*. There was a case report where this bacteria was specifically identified as a causative agent of intraocular salt; we believe that it should be studied with more interest in the future because it was resistant to both the traditional vitreous injection antibiotics vancomycin and ceftazidime.

The most isolated strains in normal conjunctiva are CNS, most of which are *S. epidermidis* [7, 26]. There were three reports of CNS antibiotic resistance in normal conjunctival flora in Korea in 1999, 2001, and 2009. Since the first published study, the other two studies reported that CNS quinolone resistance had increased compared to previous reports [5, 7]. Since this study investigated the quinolone resistance of *S. epidermidis*, it is difficult to compare 1:1 with previous CNS resistance reports; however, most of the CNS identified in the conjunctiva are *S. epidermidis*, so a rough comparison is possible. CNS susceptibility to ciprofloxacin was reported to be gradually decreased to 92.3% in 1999, 80.5% in 2001, and 69.2% in 2009, and 67.1% in this study (not statistically different from 2009). CNS susceptibility to levofloxacin was reported only in the 2009 study, and 78.7% was found to have significantly decreased susceptibility, compared to 67.1% in this study. In particular, in the 2009 report, levofloxacin sensitivity was reported at 50% for ciprofloxacin-resistant CNS strains; however, levofloxacin was also not effective for all ciprofloxacin-resistant CNS strains. The CNS susceptibility to moxifloxacin was reported only in 2009, at a rate of 89.7%, which is a significantly decreased sensitivity compared to the 74.4% in this study. Of the 20 strains that were resistant to levofloxacin, only one strain (5%) was susceptible to moxifloxacin, six strains (30%) were moderately resistant, and the remaining thirteen strains (65%) were resistant. A 2009 study reported that about 50% of levofloxacin-resistant strains showed susceptibility to 4th-generation quinolone. Based on this, it is believed that the strains showing resistance to all three quinolones increased when interpreting the results of this study. It is known that the prescription dose and resistance to antibiotics are closely related [27, 28]. As mentioned above, it is estimated that the increase in use of high-generation quinolone antibiotics may lead to an increase in *S. epidermidis*, which is resistant to all three quinolones.

In this study, QRDR mutation was observed in 20 of the 21 strains of quinolone-resistant *S. epidermidis*. The QRDR mutation pattern showed similar results compared to previous reports [18, 19, 29]. Mostly, there were mutations in *gyrA* and *parC*, and in 80 and 88 *parC* of *gyrA*, 80 and 84 of *parC* are found in this study, which is similar to the previous report. As compared with quinolone-resistant *S. epidermidis* identified in the skin, the results of this study showed a difference in the amino acid types of mutations, but the *gyrA* and *parC* mutation locations and overall patterns were similarly observed [18]. In previous *S. aureus* studies, mutations due to resistance were not observed in *gyrB*, but in the form of Ser84Leu and Glu88Lys mutations in *gyrA*, the position and pattern of *gyrA* mutations in resistant *S. epidermidis* are similar. The *grlA* and *grlB* mutations of *S. aureus* corresponding to *parC* and *parE* were similar to those of this study [15, 19].

When the number of QRDR mutations and the MIC value were correlated, the number of mutations and the MIC value tended to be proportional. However, in the case of *parE*, the number of mutations did not seem to affect the magnitude of tolerance as much as *gyrA* and *parC* (Table 6). The strains that showed the strongest resistance were those that showed multiple mutations in both *gyrA* and *parC*, and strains exhibiting this mutation are known to be rare in *S. epidermidis* [8, 9]. However, in *S. aureus*, there are reports that more than 50% of strains showed mutations in both *gyrA* and *grlA* [15].

**Table 6:** Mutations in the quinolone resistance determining region (QRDR) and minimal inhibitory concentrations (MIC, μg/mL)

Mutation type	No. of isolates	Mutation				MIC (μg/mL)		
		*gyrA*	*gyrB*	*parC*	*parE*	CFX(≥ 4)	LFX(≥ 4)	MFX(≥ 2)
1	7	Ser84Phe	–	Ser80Tyr	–	4	4	1
		Ser84Phe	–	Ser80Tyr	–	4	4	1
		Ser84Phe	-	Ser80Tyr	-	8	8	2
		Ser84Phe	-	Ser80Tyr	-	4	8	1
		Ser84Phe	-	Ser80Tyr	-	2	4	1
		Ser84Phe	-	Ser80Tyr	-	4	4	1
		Ser84Phe	-	Ser80Tyr	-	4	4	1
2	4	Ser84Phe	–	Ser80Phe	–	8	8	2
		Ser84Phe	–	Ser80Phe	–	16	8	1
		Ser84Phe	–	Ser80Phe	–	8	8	2
		Ser84Phe	–	Ser80Phe	–	8	8	2
3	3	Ser84Phe	–	Ser80Phe + Asp84Val	–	16	8	2
		Ser84Phe	–	Ser80Phe + Asp84Val	–	32	8	2
		Ser84Phe	–	Ser80Phe + Asp84Val	–	32	8	2
4	1	Ser84Tyr	–	Ser80Ile	–	32	8	2
5	1	Ser84Tyr	–	Ser80Ile	Lys402Arg + Asn404Ile	16	8	2
6	1	Ser84Phe	–	Ser80Phe + Asp84Val	Lys403Thr + Asn404Asp	16	8	2
7	1	Ser84Phe	–	Ser80Phe + Asp84Val	Lys402Arg + Lys403Arg	32	8	2
8	1	Ser84Tyr + Glu88Lys	–	Ser80Phe + Asp84Tyr	–	64	64	32
9	1	Ser84Phe	–	Ser80Phe	Asp434Val	8	8	2
10	1	–	–	–	–	2	2	8

*CFX* ciprofloxacin, *LFX* levofloxacin, *MFX* moxifloxacin

Lastly, in one strain, no mutation was observed in QRDR, but this strain was thought to exhibit quinolone resistance by mechanisms other than QRDR mutation [30], such as draining the drug out or having a resistance gene in the plasmid. In particular, this strain was sensitive to ciprofloxacin and levofloxacin of the lower generation, but showed resistance only to the highest generation moxifloxacin. Moxifloxacin is an important antibiotic in relation to surgical and severe infections in the ophthalmic area, so it would be meaningful to further study this strain in the future.

The limitation of this study was that only aerobic bacteria cultured in a nutrient medium were included in the study as a limitation of the method for culturing the bacteria. In addition, all breakpoints used here were based on systemic breakpoints provided by CLSI or derived from the method suggested by CLSI. However, the breakpoint for topical therapy has not been established so far. The concentration of antibiotic eye drops that are usually instilled in the eye is much higher than the CLSI resistance reference concentration. For this reason, it is important to investigate the resistance rate of one strain, but it is also necessary to study the changes in the superiority of the strains caused by the antibiotic drop and animal experiments later.

Studies on the normal conjunctival flora can play a very important role in understanding various ocular infectious diseases. In addition, the resistance rate and resistance gene analysis of the strain constituting the normal conjunctival flora can also provide important guidelines and data on how to use and develop antibiotics in the future. The changes in the bacterial flora and increased *S. epidermidis* resistance to 4th-generation quinolone in this study suggest that ophthalmologists prescribe a lot of higher-generation quinolone.

## Conclusions

In conclusion, this study showed the composition of normal conjunctival flora, the change in quinolone resistance rate of *S. epidermidis*, and the resistance gene mutation pattern. The ratio of *Corynebacterium* spp. among the gram-positive bacteria of the quinolone-resistant strain increased in the normal conjunctival flora, and among the gram-negative bacteria, *Ochrobactrum* spp., which was not mentioned in the previous domestic report, were cultured the most. The quinolone resistance of *S. epidermidis* increased compared to the previous domestic studies, and the strains resistant to all quinolones increased. Lastly, quinolone-resistant *S. epidermidis* showed mostly QRDR mutations, mainly in *gyrA* and *parC*, and showed the strongest resistance when both genes were mutated. These results indicate that antimicrobial stewardship is required even more in the ophthalmic area when using topical antibiotics eye drops.

## Supplementary information

**Additional file 1:** This additional file contains partial implementation, Table S1 and S2.

## Data Availability

All data generated or analyzed during this study are included in this published article and its supplementary information files.

## Abbreviations

CNS: Coagulase-negative staphylococci
QRDR: Quinolone-resistance determining region
MALDI-TOF MS: Matrix assisted laser desorption/ionization time-of-flight mass spectrometry
TSB: Tryptic soy broth
CLSI: Clinical and Laboratory Standards Institute
TSA: Tryptic soy agar
CAMHB: Cation adjusted Muller Hinton broth
MTT: 3-(4,5-Dimethylthiazol-2yl)-2,5-diphenyl tetrazolium bromide
MIC: Minimum inhibitory concentration
PCR: Polymerase chain reaction
DEPC: Diethyl pyrocarbonate
NCBI: National Center for Biotechnology Information
