# Supplementary material for: Fluoroquinolone resistance of Staphylococcus epidermidis isolated from healthy conjunctiva and analysis of their mutations in quinolone-resistance determining region
Source: Antimicrob Resist Infect Control. 2020 Nov 4;9:177. doi: 10.1186/s13756-020-00841-3 (PMC7640383; doi:10.1186/s13756-020-00841-3)
Supplement: Supplementary file 1 — Additional file 1: This additional file contains partial implementation, Table S1 and S2. [file 13756_2020_841_MOESM1_ESM.docx]

Table 1S. Results of 16S rRNA sequencing using Basic Local Alignment Search Tool (BLAST).

|  | The first matched strain | The second matched strain |
| --- | --- | --- |
| 1 | *Staphylococcus epidermidis* strain Fussel | *Staphylococcus epidermidis* strain NBRC 100911 |
| 2 | *Staphylococcus epidermidis* strain NBRC 100911 | *Staphylococcus epidermidis* strain Fussel |
| 3 | *Staphylococcus epidermidis* strain Fussel | *Staphylococcus epidermidis* strain NBRC 100911 |
| 4 | *Staphylococcus epidermidis* strain Fussel | *Staphylococcus epidermidis* strain NBRC 100911 |
| 5 | *Staphylococcus epidermidis* strain Fussel | *Staphylococcus epidermidis* strain NBRC 100911 |
| 6 | *Staphylococcus epidermidis* strain NBRC 100911 | *Staphylococcus epidermidis* strain Fussel |
| 7 | *Staphylococcus epidermidis* strain Fussel | *Staphylococcus epidermidis* strain NBRC 100911 |
| 8 | *Staphylococcus epidermidis* strain Fussel | *Staphylococcus epidermidis* strain NBRC 100911 |
| 9 | *Staphylococcus epidermidis* strain NBRC 100911 | *Staphylococcus epidermidis* strain Fussel |
| 10 | *Staphylococcus epidermidis* strain Fussel | *Staphylococcus epidermidis* strain NBRC 100911 |
| 11 | *Staphylococcus epidermidis* strain Fussel | *Staphylococcus epidermidis* strain NBRC 100911 |
| 12 | *Staphylococcus epidermidis* strain Fussel | *Staphylococcus epidermidis* strain NBRC 100911 |
| 13 | *Staphylococcus epidermidis* strain NBRC 100911 | *Staphylococcus epidermidis* strain Fussel |
| 14 | *Staphylococcus epidermidis* strain NBRC 100911 | *Staphylococcus epidermidis* strain Fussel |
| 15 | *Staphylococcus epidermidis* strain Fussel | *Staphylococcus epidermidis* strain NBRC 100911 |
| 16 | *Staphylococcus epidermidis* strain Fussel | *Staphylococcus epidermidis* strain NBRC 100911 |
| 17 | *Staphylococcus epidermidis* strain Fussel | *Staphylococcus epidermidis* strain NBRC 100911 |
| 18 | *Staphylococcus epidermidis* strain Fussel | *Staphylococcus epidermidis* strain NBRC 100911 |
| 19 | *Staphylococcus epidermidis* strain Fussel | *Staphylococcus epidermidis* strain NBRC 100911 |
| 20 | *Staphylococcus epidermidis* strain Fussel | *Staphylococcus epidermidis* strain NBRC 100911 |
| 21 | *Staphylococcus epidermidis* strain NBRC 100911 | *Staphylococcus epidermidis* strain Fussel |

Table S2. Susceptibility results of *Staphylococcus epidermidis* (n=82) to 3 fluoroquinolone antibiotics

| Micro-dilution antibiotic sensitivity test (AST) | | | | | | | | | | | | |
| --- | --- | --- | --- | --- | --- | --- | --- | --- | --- | --- | --- | --- |
| ID | CPX | LPX | MFX | ID | CPX | LPX | MFX | ID | CPX | LPX | MFX |  |
| 6-OD-1 | R | R | I | YC1-1 | S | S | S | K2-1 | S | S | S |  |
| 19-OD-1 | I | I | R | YC2-1 | S | S | S | IB1-1 | R | R | R |  |
| 20-OD-2 | R | R | R | SC1-1 | S | S | S | ZC1-2 | S | S | S |  |
| 20-OS-1 | S | S | S | ND1-1 | I | I | S | ZC2-1 | S | S | S |  |
| 22-OS-1 | R | R | R | ED2-1 | I | I | S | P2-1 | S | S | S |  |
| 23-OD-2 | S | S | S | IC2-2 | S | S | S | P2-2 | S | S | S |  |
| 28-OS-1 | S | S | S | DD1-1 | S | S | S | MD2-1 | S | S | S |  |
| 31-OS-1 | S | S | S | DD2-1 | I | I | S | GD1-1 | R | R | R |  |
| 32-OS-1 | R | R | I | UA2-1 | S | S | S | GD2-1 | S | S | S |  |
| 36-OD-1 | R | R | R | BB1-1 | R | R | R | LD2-1 | S | S | S |  |
| 38-OS-1 | S | S | S | VA2-1 | S | S | S | RC2-1 | S | S | S |  |
| 42-OD-1 | S | S | S | QA2-1 | S | S | S | KD1-1 | S | S | S |  |
| 43-OD-2 | S | S | S | UC1-2 | S | S | S | KD2-1 | S | S | S |  |
| 43-OS-1 | I | I | S | UC2-1 | I | R | I | O1-1 | S | S | S |  |
| 49-OS-1 | S | S | S | LC 2-1 | R | R | R | XC1-1 | S | S | S |  |
| 60-OS-1 | R | R | S | LC 2-2 | R | R | R | XC2-1 | S | S | S |  |
| 61-OS-1 | R | R | I | H 2-1 | S | S | S | YD1-1 | R | R | I |  |
| 63-OS-1 | S | S | S | G 2-1 | S | S | S | ZD1-1 | R | R | R |  |
| 66-OS-1 | S | S | S | CC 1-1 | S | S | S | BE2-1 | S | S | S |  |
| AC 1-1 | I | I | S | CC 1-2 | S | S | S | EE1-1 | R | R | R |  |
| HD 1-1 | R | R | R | DC 1-1 | S | S | S | EE1-2 | I | I | I |  |
| RB 2-1 | S | S | S | DC 1-2 | S | S | S | FE2-1 | S | S | S |  |
| TB 2-1 | S | S | S | VC 1-1 | S | S | S | GE101 | S | S | S |  |
| KC 1-1 | S | S | S | VC 2-1 | S | S | S | HE2-2 | S | S | S |  |
| KC 2-1 | S | S | S | EB 1-1 | S | S | S | JE101 | R | R | R |  |
| LC 1-1 | R | R | R | VB 2-1 | S | S | S | LE2-1 | R | R | I |  |
| OB1-2 | S | S | S | VB 2-2 | S | S | S | OE2-1 | S | S | S |  |
| OB2-2 | S | S | S |  |  |  |  |  |  |  |  |  |

S: susceptible, I: intermediated resistance, R: resistant, CPX: ciprofloxacin, LPX: levofloxacin, MFX: moxifloxacin
